# Supplementary material for: Immunoregulatory Biomarkers of the Remission Phase in Type 1 Diabetes: miR-30d-5p Modulates PD-1 Expression and Regulatory T Cell Expansion
Source: Noncoding RNA. 2023 Feb 28;9(2):17. doi: 10.3390/ncrna9020017 (PMC10037622; doi:10.3390/ncrna9020017)
Supplement: Supplementary file 1 [file ncrna-09-00017-s001.zip › ncrna-2218879-supplementary.pdf]

Supplementary Data

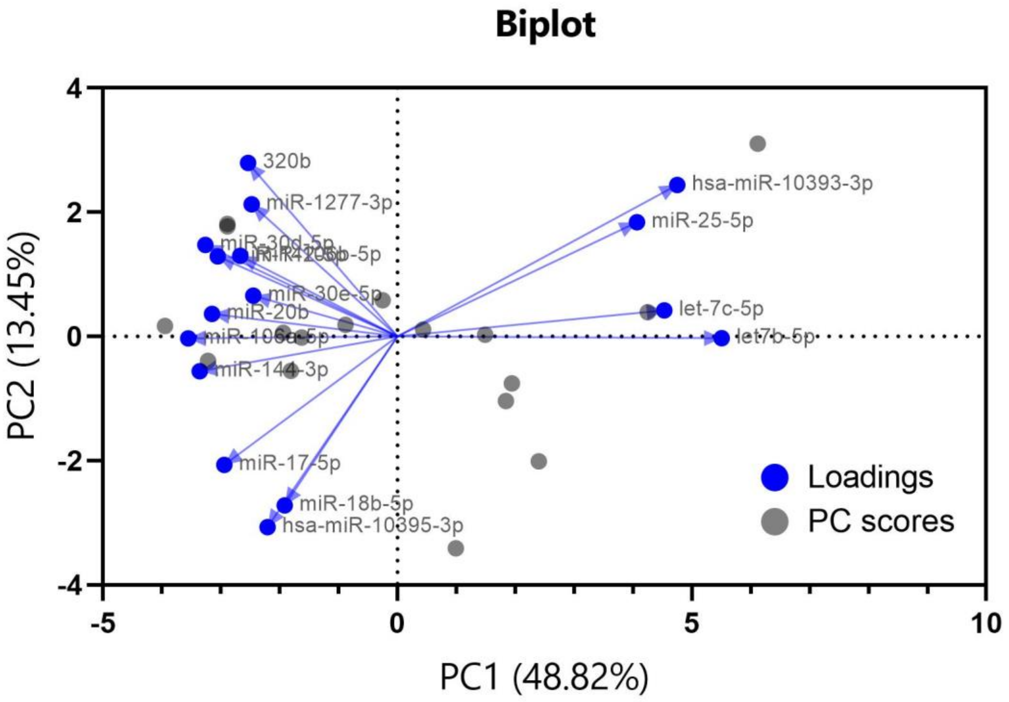

Figure S1. **Biplot of the principal component analysis (PCA) for differentially expressed miRNAs during the partial remission phase.** The two principal components (PC1 and PC2) explained 62.27% of the total variation in miRNA data and shows clear partitioning of PR and non-PR samples along the PC1. let-7b-5p and let-7c-5p were the miRNAs influencing the most the non-PR group. Blue arrows represent the differentially expressed miRNAs, and circles in grey represent sampling points (PR and non-PR).

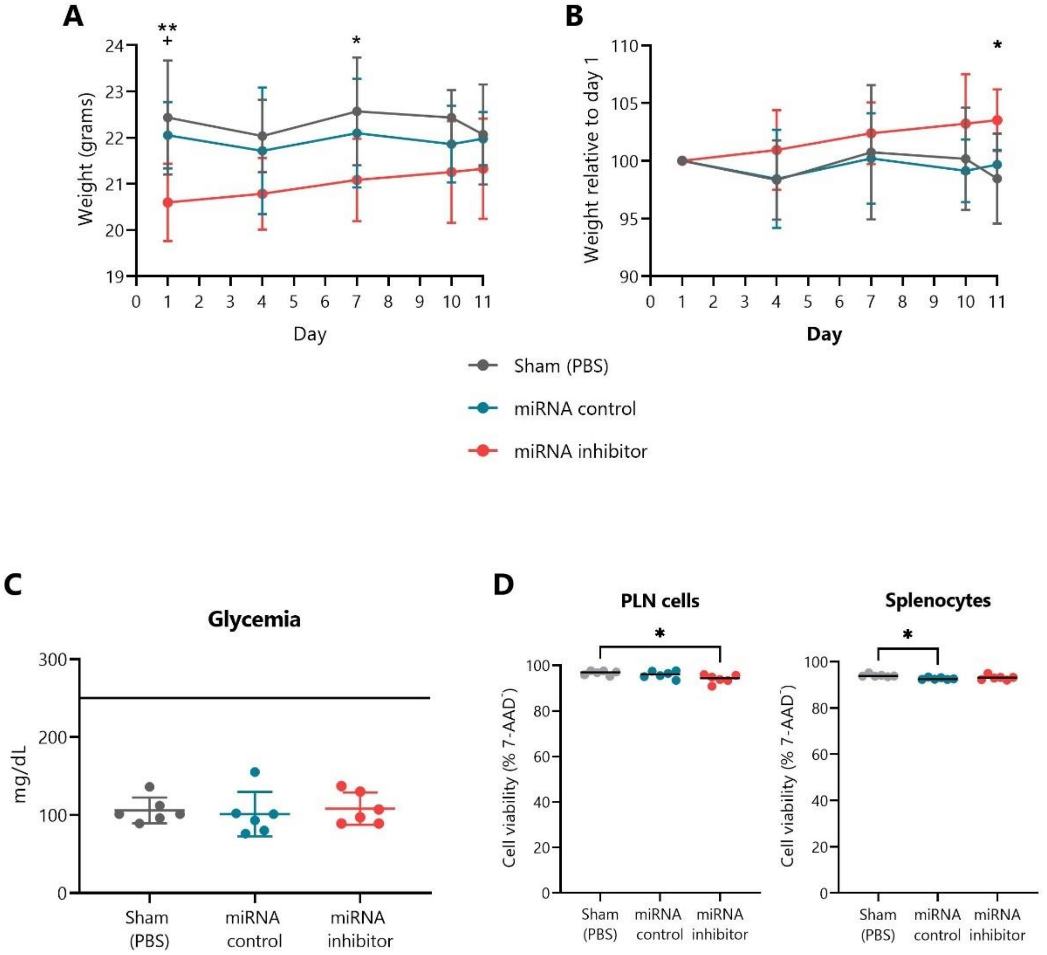

Figure S2. **Normal weight and glycemia values, and optimal cell viability after the treatment with miR-30d-5p inhibitor.** (**A**) Weight measured through the treatment period at days 1 (pre-treatment), 4, 7, 10 and 11 (24h after the last injection). (**B**) Weight relative to day 1 (pretreatment) measured at days 4, 7, 10 and 11 (24h after the last injection). Grey lines and dots correspond to the sham group, blue lines and dots to the control inhibitor group, and red lines and dots to the miR-30d-5p inhibitor group. *p ≤0.05 **p ≤0.01 (inhibitor vs sham), $^{+}$p ≤0.05 (inhibitor vs control), 2way ANOVA. (**C**) Blood glucose levels (mg/dL) after the short treatment, measured at day 11 (24h after the last injection) in sham mice, mice treated with a control inhibitor, or mice treated with the miR-30d-5p inhibitor. Data are presented as mean±SD. The black line indicates the level from which hyperglycemia is considered in mice. Significant differences were not found between groups. (**D**) Viability of PLN cells and splenocytes from sham mice, mice treated with a control inhibitor, or mice treated with the miR-30d-5p inhibitor assessed by 7aad staining. Black lines indicate the median percentage of cell viability. *p ≤0.05, Kruskal-Wallis with Dunn's post-hoc test. n=6 per group.

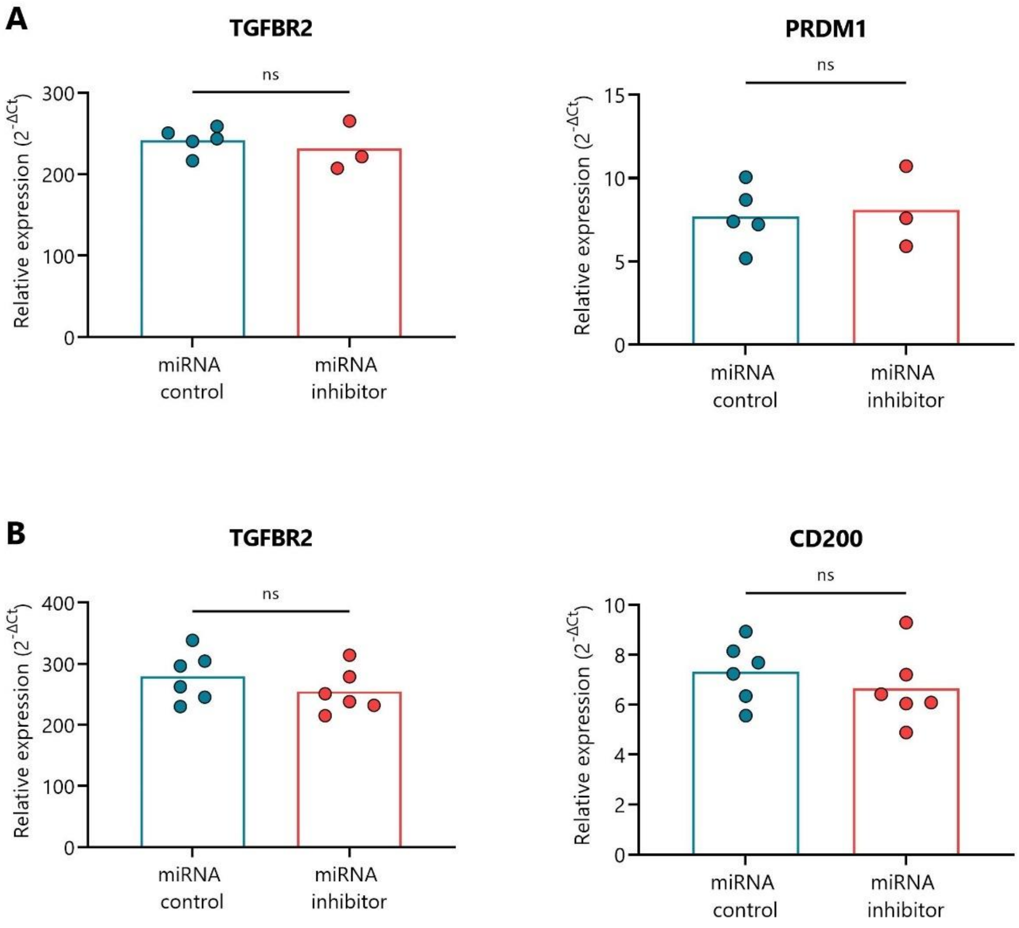

Figure S3. **Gene expression levels of TGFBR2, PRDM1, and CD200.** (**A**) Levels of TGFBR2 and PRDM1 mRNAs on the remaining PLN cells of mice treated with a control inhibitor (n=5, left, blue) or the miR-30d-5p inhibitor (n=3, right, red). (**B**) Levels of TGFBR2 and CD200 mRNAs on the remaining splenocytes of mice treated with a control inhibitor (n=6, left, blue) or the miR-30d-5p inhibitor (n=6, right, red). Gene expression signal was normalized to *GAPDH* expression. Values are expressed as $2^{-\Delta Ct}$. Data are presented as mean±SD. Significant differences were not found between groups.

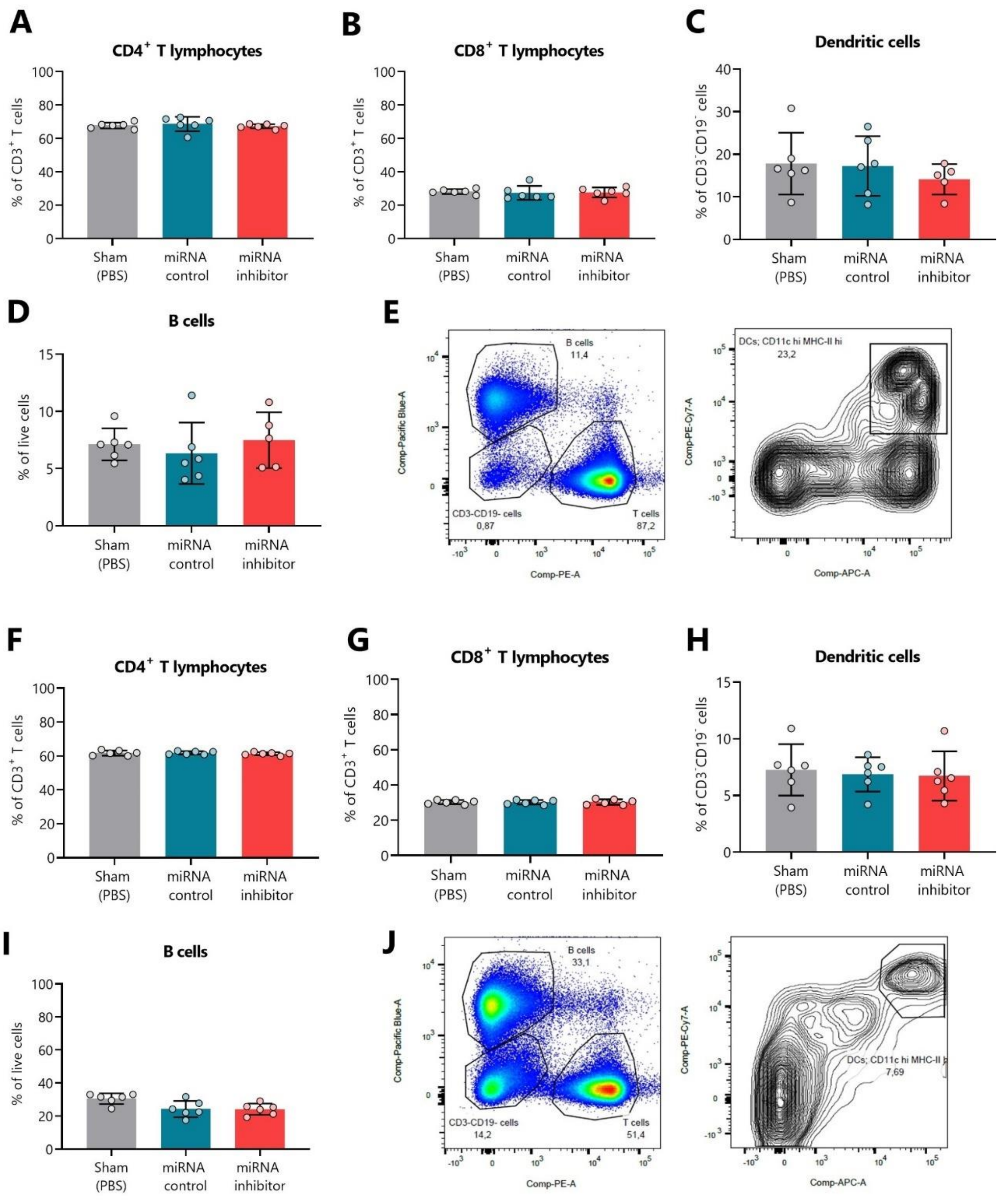

Figure S4. **miR-30d-5p inhibition does not affect the percentages of total CD4$^+$ and CD8$^+$ T cells, B cells, or dendritic cells.** (**A-D and F-I**) Percentages of CD4$^+$ T cells, CD8$^+$ T cells, dendritic cells, and B cells in (**A-D**) PLN or (**F-I**) the spleen of sham mice (n=6, left, grey), mice treated with a control inhibitor (n=6, middle, blue) or mice treated with the miR-30d-5p inhibitor (n=5–6, right, red). Data are presented as mean±SD. Significant differences were not found between groups. (**E and J**) Representative gating strategy for B cells, T cells, and DCs (from CD3$^-$CD19$^-$ cells) in (**E**) PLN and (**J**) the spleen.

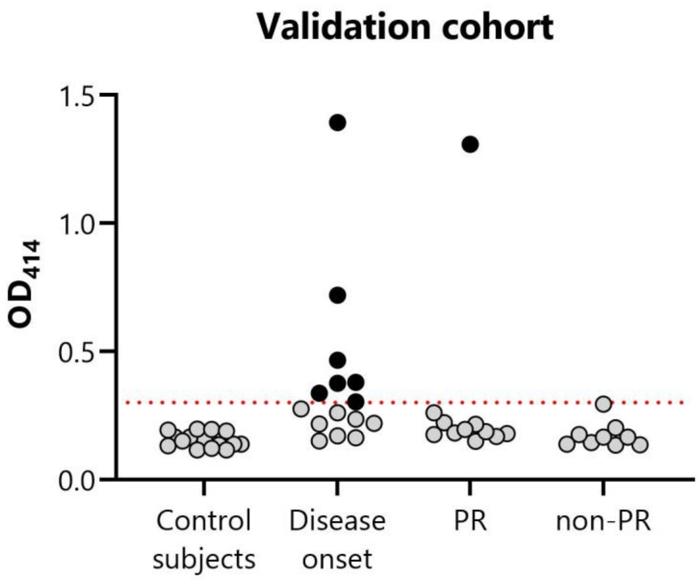

Figure S5. **Analysis of the hemolysis in plasma samples**. The degree of hemolysis was checked in control subjects (n=15), patients at disease onset (n=15), at PR (n=11), or non-PR (n=9) by measuring the optical density at 414 nm (absorbance peak of free hemoglobin) by spectrophotometry. The severely hemolyzed samples (OD_{414} >0.3) were discarded. Black dots represent discarded samples and grey dots the samples used in further experiments. The red discontinuous line indicates the OD_{414} =0.3.

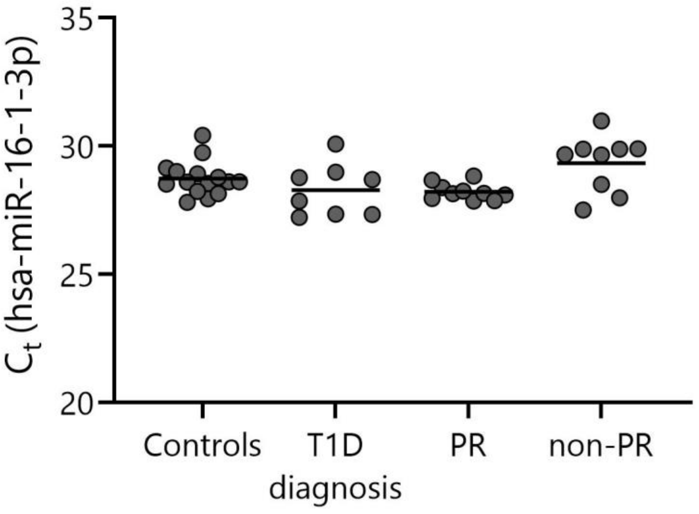

Figure S6. **Raw cycle threshold (C_t) values of miR-16-1-3p.** The raw C_t values of miR-16-1-3p were obtained by RT-qPCR in plasma samples of controls (n=15), patients at T1D diagnosis (n=8), at PR (n=10), and non-PR (n=9). No differences were found between the different groups (by Kruskal-Wallis with Dunn's post-hoc test).

Table S1. **Differentially expressed circulating miRNAs with validated target genes during the partial remission phase or at T1D diagnosis versus nondiabetic controls.**

**miRNAs**	**PR vs Ctrl (FC)**	P-value	**T1D dx vs Crtl (FC)**	P-value
hsa-miR-3173-5p	-2.491	0.001	-	-
hsa-miR-21-3p	-2.415	0.001	-	-
hsa-miR-940	-2.463	0.001	-	-
hsa-miR-769-5p	-2.322	0.002	-	-
hsa-miR-205-5p	-2.197	0.003	-	-
hsa-miR-30e-3p	2.316	0.003	-	-
hsa-miR-4433b-5p	-2.241	0.004	-	-
hsa-miR-424-5p	1.852	0.004	-	-
hsa-miR-3138	-2.293	0.004	-	-
hsa-miR-133a-3p	-2.059	0.005	-	-
hsa-miR-362-5p	-2.098	0.005	-	-
hsa-miR-410-3p	-2.178	0.005	-	-
hsa-miR-3200-5p	-2.546	0.005	-	-
hsa-miR-424-3p	-2.178	0.006	-	-
hsa-miR-3202	-2.154	0.006	-	-
hsa-miR-203a-3p	-2.013	0.007	-	-
hsa-miR-194-5p	2.015	0.008	-	-
hsa-miR-190a-5p	2.044	0.009	-	-
hsa-miR-26a-2-3p	-2.080	0.009	-	-
hsa-miR-23a-5p	-1.968	0.010	-	-
hsa-miR-338-3p	1.942	0.010	2.299	0.007
hsa-miR-92b-5p	-1.851	0.014	-	-
hsa-miR-33b-5p	-1.922	0.015	-	-
hsa-miR-432-5p	-1.800	0.015	-	-
hsa-miR-655-3p	-1.801	0.015	-	-
hsa-miR-877-5p	-2.106	0.016	-	-
hsa-miR-580-3p	-1.823	0.017	-	-
hsa-miR-429	-1.819	0.018	-	-
hsa-miR-1307-3p	-1.836	0.018	-	-
hsa-miR-455-5p	-1.798	0.019	-	-
hsa-miR-106b-3p	1.600	0.020	-	-
hsa-miR-130b-5p	-1.709	0.020	-	-
hsa-miR-6891-3p	-1.718	0.021	-	-
hsa-miR-181a-3p	-1.764	0.022	-	-
hsa-miR-6503-5p	-1.697	0.022	-	-
hsa-miR-3613-5p	1.647	0.022	-	-
hsa-miR-152-3p	-1.904	0.023	-	-
hsa-miR-103a-3p	1.729	0.023	-	-
hsa-miR-495-5p	-1.786	0.024	-	-
hsa-miR-6734-5p	-1.761	0.024	-	-
hsa-miR-556-3p	-1.783	0.026	-	-
hsa-miR-331-3p	-1.767	0.027	-	-
hsa-miR-26a-1-3p	-1.671	0.027	-	-
hsa-miR-3200-3p	-1.666	0.027	-	-
hsa-let-7g-3p	-1.645	0.027	-	-
hsa-miR-376a-3p	-1.695	0.028	-	-

hsa-miR-495-3p	-1.686	0.028	-	-
hsa-miR-1908-5p	-1.639	0.029	-	-
hsa-miR-454-3p	1.292	0.032	-	-
hsa-miR-186-5p	1.422	0.032	-	-
hsa-miR-218-5p	-1.643	0.032	-	-
hsa-miR-4484	-1.711	0.033	-	-
hsa-miR-144-5p	1.059	0.035	-	-
hsa-miR-1301-3p	-1.579	0.036	-	-
hsa-miR-3179	-1.590	0.039	-	-
hsa-miR-16-2-3p	1.372	0.040	-	-
hsa-miR-570-3p	-1.531	0.041	-	-
hsa-miR-501-3p	-1.526	0.043	-	-
hsa-miR-374a-5p	1.265	0.044	-	-
hsa-miR-378a-3p	-1.676	0.045	-	-
hsa-miR-411-3p	-1.553	0.048	-	-
hsa-miR-107	1.522	0.049	-	-
hsa-miR-144-3p	-	-	1.425	0.0004
hsa-miR-130a-3p	-	-	1.456	0.011
hsa-miR-17-5p	-	-	1.272	0.015
hsa-miR-106b-3p	-	-	1.813	0.020
hsa-miR-10b-5p	-	-	-2.170	0.022
hsa-miR-140-5p	-	-	-2.164	0.024
hsa-miR-20a-5p	-	-	1.045	0.032
hsa-miR-3648	-	-	-1.732	0.033
hsa-miR-1277-3p	-	-	1.957	0.035
hsa-miR-223-3p	-	-	-1.189	0.036
hsa-miR-183-5p	-	-	1.712	0.041
hsa-miR-197-3p	-	-	-1.876	0.043
hsa-miR-582-5p	-	-	-1.675	0.045
hsa-miR-22-3p	-	-	1.740	0.047
hsa-miR-191-5p	-	-	1.123	0.049

Data analyzed by Moderated *t*-test. *Abbreviations*: *Ctrl*, controls; *FC*; fold change; *PR*, partial remission; *T1D dx*, type 1 diabetes diagnosis.

Table S2. **Classification of some of the miR-30d-5p target genes into regeneration, metabolism, and immune system groups.**

miRNA	Genes for regeneration	Genes for metabolism	Genes for immune system
hsa-miR-30d-5p	ADAM9, ADGRE1, APPL1, BCL11A, BNIP3, BNIP3L, CASP4, CASP7, DEGS1, DIDO1, FAT1, GXYLT1, MCL1, NOTCH1, NOTCH2, NRP1, NRXN3, RAB11FIP1, RBBP6, TNC, UBN1, VEGFA, VEGFC, ZFP36L1, ZFP36L2	CPEB2, DDX6, EDC3, HSPA5, IREB2, MYCBP2, OAS1, OAS2, RIOX2, VKORC1L1, XRN1	ATG12, BCL6, BMP2, CCL17, CCL5, CCR1, **CD200**, CD226, CNOT9, CTLA4, CXCL11, CXCL8, DIDO1, FAF2, FAS, FCRLB, FMOD, GCSAM, GDNF, HLA-DRA, IFITM1, IFITM2, IFITM3, IL10, IL1RAPL1, IL21R, INHBA, ITSN2, JAK1, KLF14, LAMP3, LGR4, MAPK8, MICB, NCR3LG1, NFAT5, NFATC3, NMI, OTUD6B, **PRDM1**, PTPN13, SKI, SMAD2, SMAD7, SOCS1, STON1, TAF4B, **TGFBR2**, TGFBR3, TNFSF13B, TNFSF9
